# Enhancing knowledge discovery from unstructured data using a deep learning approach to support subsurface modeling predictions

**DOI:** 10.3389/fdata.2023.1227189

**Published:** 2023-12-19

**Authors:** Brendan Hoover, Dakota Zaengle, MacKenzie Mark-Moser, Patrick Wingo, Anuj Suhag, Kelly Rose

**Affiliations:** ^1^National Energy Technology Laboratory, Albany, OR, United States; ^2^NETL Support Contractor, Albany, OR, United States; ^3^US Army Corps of Engineers, Geospatial Research Laboratory, Alexandria, VA, United States

**Keywords:** subsurface, knowledge discovery (data mining), unstructured data, deep learning, artificial intelligence, modeling

## Abstract

Subsurface interpretations and models rely on knowledge from subject matter experts who utilize unstructured information from images, maps, cross sections, and other products to provide context to measured data (e. g., cores, well logs, seismic surveys). To enhance such knowledge discovery, we advanced the National Energy Technology Laboratory's (NETL) Subsurface Trend Analysis (STA) workflow with an artificial intelligence (AI) deep learning approach for image embedding. NETL's STA method offers a validated science-based approach of combining geologic systems knowledge, statistical modeling, and datasets to improve predictions of subsurface properties. The STA image embedding tool quickly extracts images from unstructured knowledge products like publications, maps, websites, and presentations; categorically labels the images; and creates a repository for geologic domain postulation. Via a case study on geographic and subsurface literature of the Gulf of Mexico (GOM), results show the STA image embedding tool extracts images and correctly labels them with ~90 to ~95% accuracy.

## 1 Introduction

Artificial Intelligence (AI) shines in its capacity to perform intricate calculations and uncover hidden patterns that surpass human capabilities (Zhan et al., 2023). This proficiency finds notable application in geospecific research, particularly in the development of binary and multimodal classification algorithms for subsurface analysis, effectively categorizing features like reservoirs, aquifers, faults, and fractures by processing visual data, including images, maps, and cross sections (Zhan et al., 2023).

AI also plays a pivotal role in advancing deep learning algorithms for seismic data interpretation, exceeding traditional methods in precision and efficiency (Tschannen, 2020). This heightened accuracy not only results in more precise geological models of the subsurface but also reinforces AI's potential to reshape the field of geoscientific research.

However, pattern recognition's role extends far beyond result-based algorithms; it is a fundamental element that permeates the entire research process. This significance becomes notably pronounced during the literature review phase, where researchers navigate through extensive volumes of unstructured data (Wagner et al., 2022). Already, AI's proficiency in rapid data parsing provides invaluable assistance to researchers during this process, effectively contributing to the identification and validation of research gaps, the execution of repetitive tasks, the extraction of metadata, and the facilitation of qualitative content analysis (Wagner et al., 2022).

The term “literature review” may conjure images of scientists immersed in volumes of books, manuscripts, and densely written material. However, in practice, the visual content within these documents often facilitates knowledge acquisition as effectively, if not more so, than written text. This dynamic is particularly prominent in fields such as geography and geology. For example, researchers can understand in seconds the distribution of fluvial systems that deposit into the Gulf of Mexico when looking at a map; a text description of where these fluvial systems are located would require extreme detail or prior understanding of the geography to comprehend their location.

**Figure 1:**
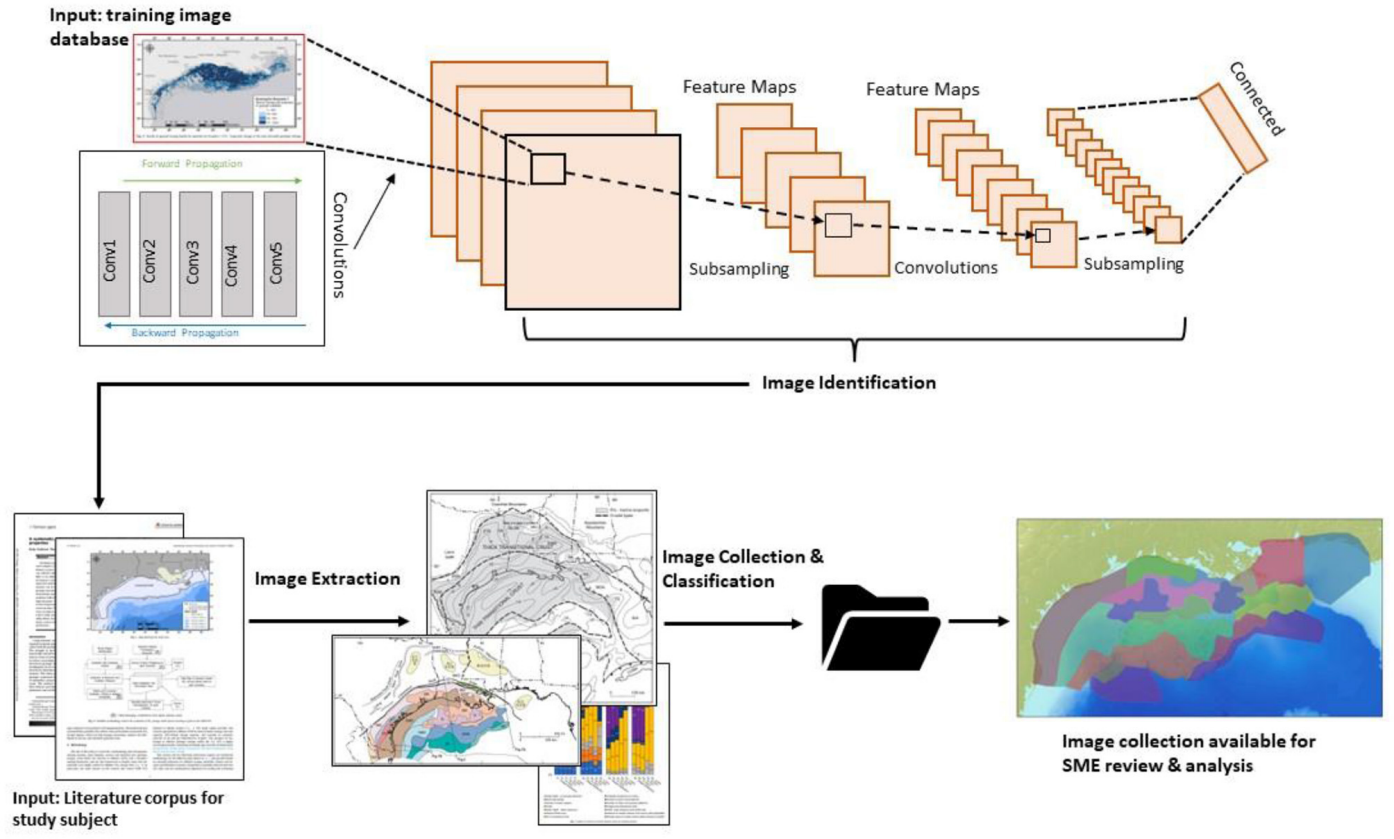
Visualization of NETL's STA Image Imbedding Tool. At top, the model was trained on image data consistent with geospecific research. The tool then extracts imagery from literature, labels, collects/organizes the imagery, and makes the data available for review and analysis.

Given the value of visualization to knowledge transfer in earth sciences, like geography and geology, and the power of AI computer vision in image-pattern recognition, this paper introduces an AI-informed image segmentation/imbedding tool, which parses and categorizes unstructured knowledge products like images, maps, cross sections, and other visualizations from geoscientific and related literature. The image embedding tool allows users to input literature from their local machine (singularly or in batches) or input a list of internet links. In either instance, the tool extracts visualizations, categorizes the visualization via a convolutional neural net (CNN) built on the VGG16 architecture, and gives the user the opportunity to view them (Figure 1). The image imbedding tool is part of a software suite that emerged from the National Energy Technology Lab's (NETL) Subsurface Trend Analysis (STA) workflow, which was developed to assist subsurface research by bringing greater contextual knowledge to measured data such as cores, well logs, and seismic surveys (Rose et al., 2020).

## 2 Methods

### 2.1 The subsurface trend analysis method

The STA method is a theoretical foundation proposed by NETL to enhance predictions of subsurface properties via the integration of geologic context with geo- and spatial statistics (Rose et al., 2020). Building upon that theoretical foundation, NETL developed an STA software suite to integrate AI/ML with the STA method; the image imbedding tool showcased in this paper is one part of that broader tool (Mark-Moser et al., in prep). The STA method follows four steps to achieve this integration: (1) gathering of geologic knowledge and subsurface data, (2) postulating geologic domains, (3) validating geologic domain, and (4) conducting advanced analyses that integrate geologic domains. These first two steps involve a literature review to identify geologic information (e.g., geologic maps, provinces, and stratigraphy) that can be used to postulate geologic domains that bound areas of common geology relative to the subsurface property the user investigates. In support of steps 1 and 2, the deep learning technique of image embedding was selected because it can harvest visual information on geologic and geographic context from publications while reducing oversight and shortening the user's review time.

### 2.2 Image imbedding

The STA image embedding tool uses a modified version of Python's Fitz library for image extraction (McKie and Liu, 2021), then categorically labels those extracted images via an expanded convolutional neural net (CNN) built upon the VGG16 architecture (Ranjan, 2020). CNNs use image segmentation to partition imagery into perceptual or visual regions, based on pixel values and pattern recognition (Wang et al., 2018). Pixels with similar values and patterns belong to the same object, parts of objects, or to the background, which typically have smaller feature differences. The pixel values are based on an ensemble of color, textures, gradients, and light intensity. Consistent patterns of pixels are grouped together as belonging to an object.

**Figure 2:**
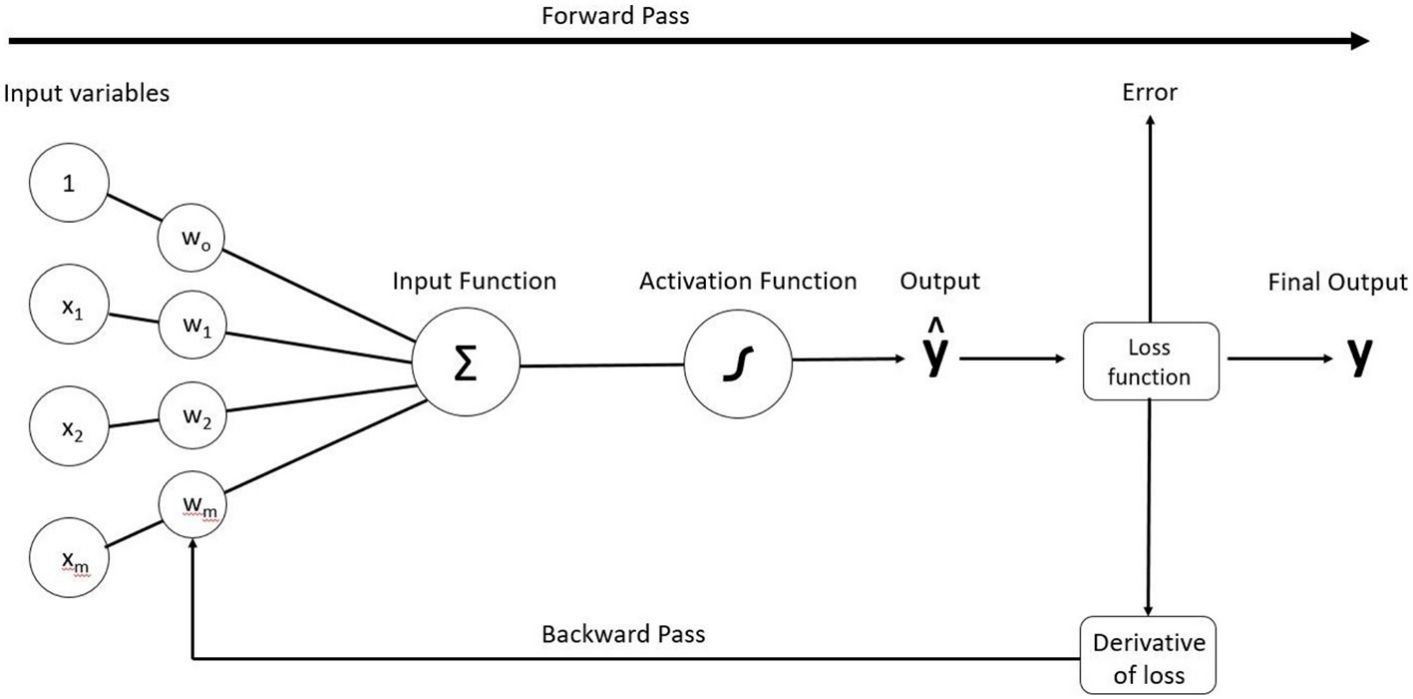
Visualization of CNN. An epoch is a complete forward pass and backward pass, which adjusts parameter weights until error is minimized for the final output. An epoch can take place with an entire dataset or with batches.

Theoretically, in a process referred to as computer vision, the CNN is meant to mimic the human eye, which seamlessly uses attributes like color or texture to partition imagery into an almost infinite number of categories. In practice, a CNN model “reviews” training data or thousands of pre-categorized images and over time begins to recognize patterns, a process referred to as model training (Wang et al., 2018). Like human learning, the accuracy of the pattern recognition relies on repetition, so the model reviews the training data several times. In the parlance of deep learning, the number of times that a neural net reviews an entire training dataset and accordingly adjusts the model is referred to as an epoch. In practice, an epoch is also broken into batches, which updates the model before the entire dataset is viewed (Kattenborn et al., 2021). At the end of a batch, the predicted variables are compared to the expected output allowing for error measurement via a loss function (forward pass) and an adjustment (backward pass) to model weights, which is a measure of the importance of each input parameter (Figure 2). It is expected that error will decrease and accuracy will increase during each epoch as the model trains. However, there will eventually be a stabilization of error and accuracy (i.e., convergence), at which point running more epochs will have diminishing returns and an increased potential for overfitting (Gavrilov et al., 2018).

While humans can easily recognize small perceptual differences in objects or images, computer recognition, to date, is much less advanced. For example, humans can easily recognize differences in dog breeds, but at present, computers struggle to ascertain that an image of a husky and pug would be categorized as the same species (i.e., as a dog) (Moreira et al., 2017). Despite limitations, with the advent of ever faster processing speeds, and greater amounts of storage and data, the capabilities of computer vision have grown exponentially during the past decade (Bayoudh et al., 2022). In fact, computer vision technology is now part of daily life, particularly for yes-or-no decisions. For instance, Apple's iPhone uses binary facial recognition to recognize phone owners vs. non-owners. In a major world health development, computer vision is being used to distinguish the presence of malaria in blood samples (Pattanaik et al., 2017).

### 2.3 Model development

**Figure 3:**
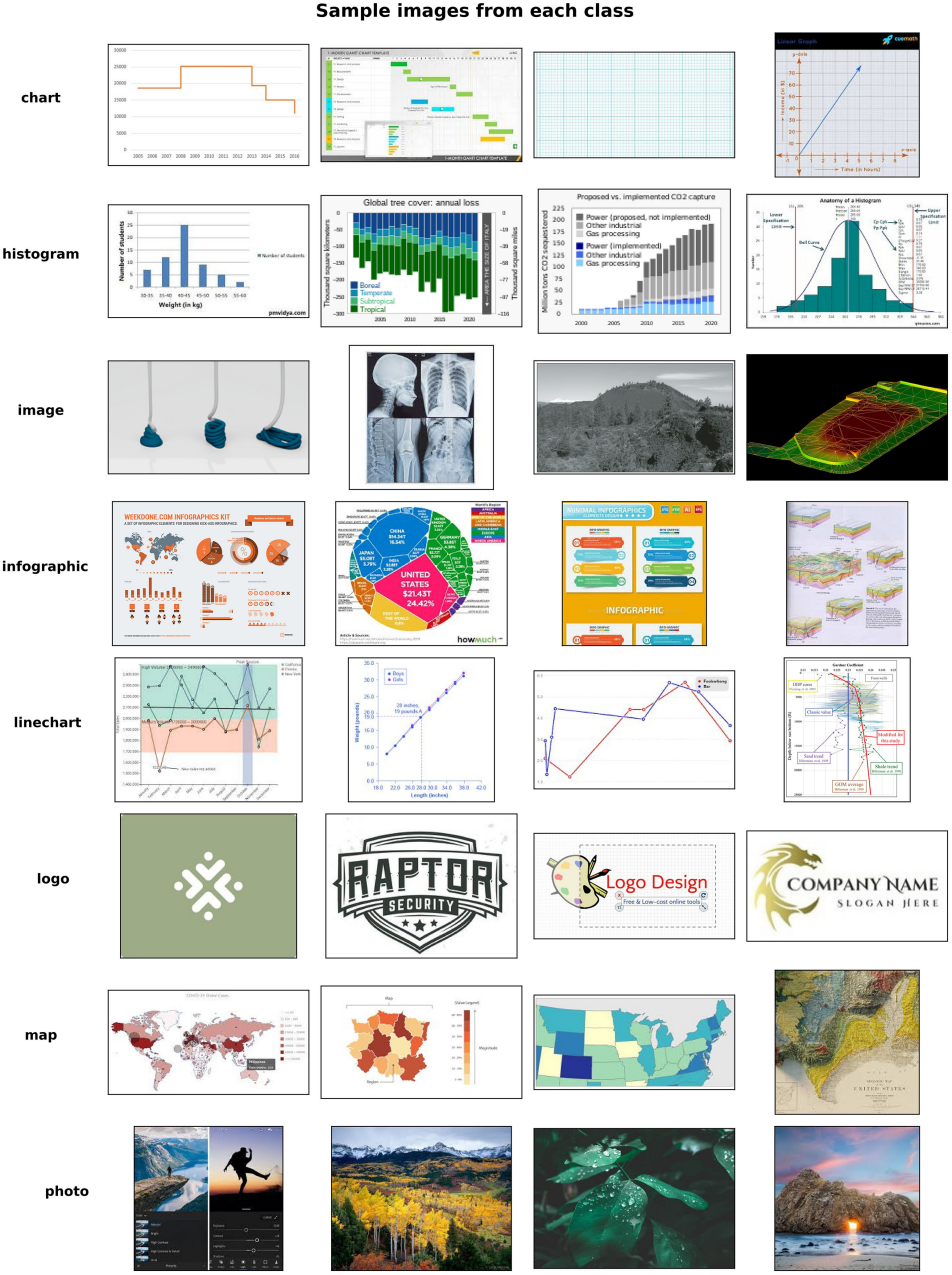
Visualization of multi-category training data for STA image embedding tool.

In computer vision research, it is common to initiate the modeling process by creating models capable of distinguishing between two categories, for instance, differentiating between cats and dogs. These models are subsequently extended to encompass broader categories, such as specific breeds within the cat or dog category (Khalifa et al., 2022). We adopted a similar approach in the development of the NETL STA image embedding tool. Given the focus of our research on geospatial and geologic data, our initial objective was to design a model with the ability to classify images into one of two primary geospecific data types, namely maps or charts. Our model was then further extended to include additional categories, as seen in Figure 3.

Furthermore, in the realm of computer vision, transfer learning is a widely adopted machine learning strategy. This technique involves using a model originally designed for one specific task as the foundational basis for a model intended for a different task. This can be useful when the second task is like the first task, but there is less data available to train a model from scratch (Pan and Yang, 2009). We evaluated various pre-existing models, including ResNet50, VGG16, VGG1, Inceptionv3, and EfficientNet (Ranjan, 2020). Among these, the VGG16 model, readily accessible in numerous software packages like TensorFlow, Keras, or PyTorch (Ranjan, 2020), stood out as the most effective in initial tests. VGG16 was developed by Karen Simonyan and Andrew Zisserman at the University of Oxford in 2014 (Simonyan and Zisserman, 2014). The VGG16 model has 16 convolutional layers and three fully connected layers (Yosinski et al., 2014). While other models demonstrated slight improvements of ~10–15% above chance levels, the VGG16 model outperformed them. It's worth noting, however, that even though VGG16 showed superior performance compared to other pre-built models, it still achieved a classification accuracy of only around 20% above chance when distinguishing between maps and charts.

**Figure 4:**
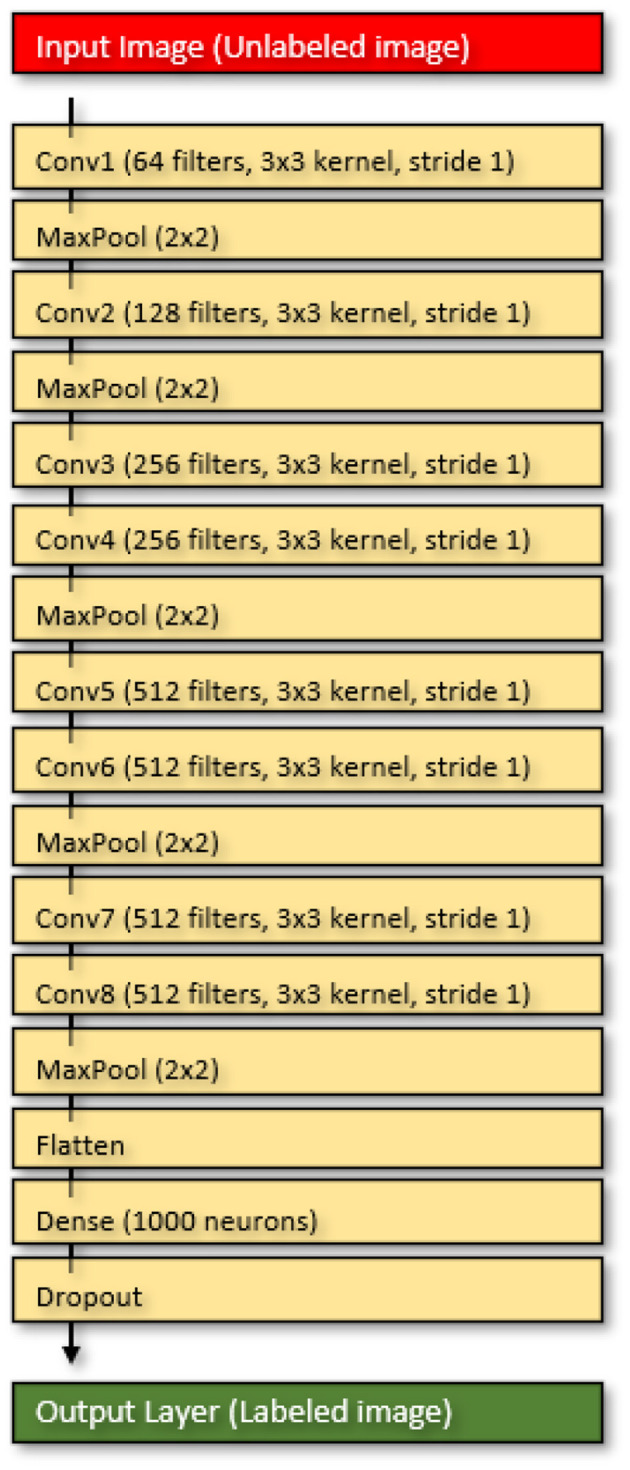
The neural network layers of the STA Image Imbedding tool.

To enhance the accuracy of the VGG16 model for our STA image embedding tool, we introduced additional sequential layers to the Convolutional Neural Network (CNN). These included dense layers, flattening, and dropout (Figure 4). We also applied the appropriate loss functions based on the number of modeled classes, utilizing binary-cross entropy for binary classification and categorical-cross entropy for multi-categorical models. In our quest to refine the VGG16 model, we followed standard deep learning model development practices, drawing inspiration from Ranjan (2020). Nevertheless, it's important to note that creating our model was an iterative process, requiring adjustments to both layers and hyperparameters until we achieved optimal accuracy while preventing over-tuning by minimizing cross-entropy (Srivastava et al., 2014; Goodfellow et al., 2016).

For clarity, dense layers refer to fully connected layers, meaning that each neuron in the layer is connected to every neuron in the previous layer. These layers are typically used for classification tasks (Goodfellow et al., 2016). Flattening, on the other hand, involves converting the output of a convolutional layer into a one-dimensional vector, which is necessary before passing it to a fully connected layer (LeCun et al., 2015). Dropout is a technique utilized to prevent overfitting in neural networks by randomly deactivating neurons during training. This encourages the network to learn from multiple features rather than relying on just a few (Srivastava et al., 2014). In our model, the input image initially goes through the VGG16 model, and the output is then flattened into a one-dimensional vector. This vector is subsequently processed through a series of STA Image embedding dense layers, followed by a dropout layer (Figure 4). The final output layer contains one neuron for each class, with the class having the highest output value being the predicted outcome.

### 2.4 Model categories

A key component to deep learning model development is the training data. We began collecting training data with a web-scraper and labeled images manually as map or chart. Initial accuracy was poor, so we fine-tuned both the training data (collecting more specific maps from scientific literature) and fine-tuned the CNN model itself.

Multi-classes were defined to capture common figures in literature, balanced between specificity and the model's ability to differentiate imagery. Table 1 provides a short description of each class and a breakdown of each dataset size. The dataset size varies for a number of reasons:

**Table 1:** Description of model categories.

**Class**	**Description**	**Train/validation/test set size**
Chart	Acts as a catch all for data representations that are not line charts, tables, or histogram/bar charts. Dataset includes many examples of pie charts, gantt charts, organizational charts, scatter plots. This class came from charts that were not common enough to create their own class.	164/15/6
Histogram/bar chart	Data representation containing clearly defined bars representing categorical or binned continuous data.	151/18/14
Photo	A standard RGB photograph taken from a consumer camera with minimal editing.	399/31/12
Image	Different from “Photo” this is expected to capture figures beyond what a regular camera would produce. Examples include 3D renders and electron microscope scans.	38/14/13
Infographic	Information displayed as multiple objects, may contain examples of other classes.	122/12/17
Line chart	Data displayed using connected line segments.	151/20/29
Logo	A graphic that may contain text representing an organization, method, or tool.	266/32/14
Map	A display expected to contain some geospatial boundary.	275/27/18
Stratigraphy/cross section	A graphic containing subsurface and/or geologic information at depth.	563/55/16
Table	Data displayed in cells, ideally with defined borders around each cell, in even rows and columns.	380/33/10
Total		2,509/257/149

(1) some classes were combined to better represent visual differences, (2) the results of the google image search changed in size and quality, and (3) some classes had many duplicates that were removed upon inspection.

### 2.5 Testing the model

To train the STA image embedding CNN, we split the data into three categories: (1) training, (2) validation, and (3) testing. This splitting process is typically automated, but we selected the test dataset by hand to ensure there were no similar or duplicated images from the training and validation sets. The division of the training, validation, and testing set was ~85/7.5/7.5%, respectively, except for some classes where the test set was kept closer to the size of the less represented classes. From the training and validation results, we found that 20 epochs balanced the variance and bias trade off (i.e., avoiding overfitting). Following training, we used the unseen test set for accuracy assessment.

### 2.6 Deploying the model

**Figure 5:**
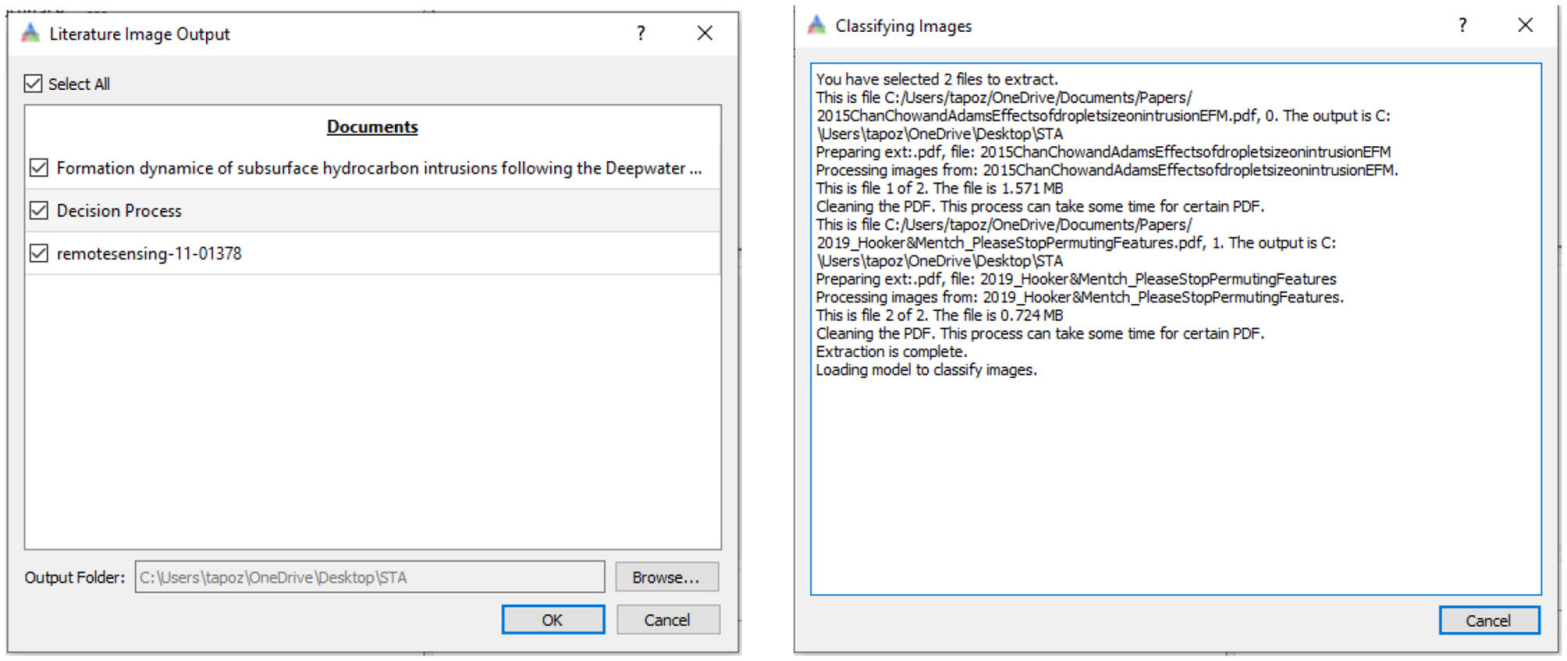
User interfaces for the STA image embedding tool: the image embedding UI allows users to import a list of online or local knowledge products **(left)** to create a repository of structured data to assist in domain postulation. Showing progress as images are labeled **(right)**.

**Figure 6:**
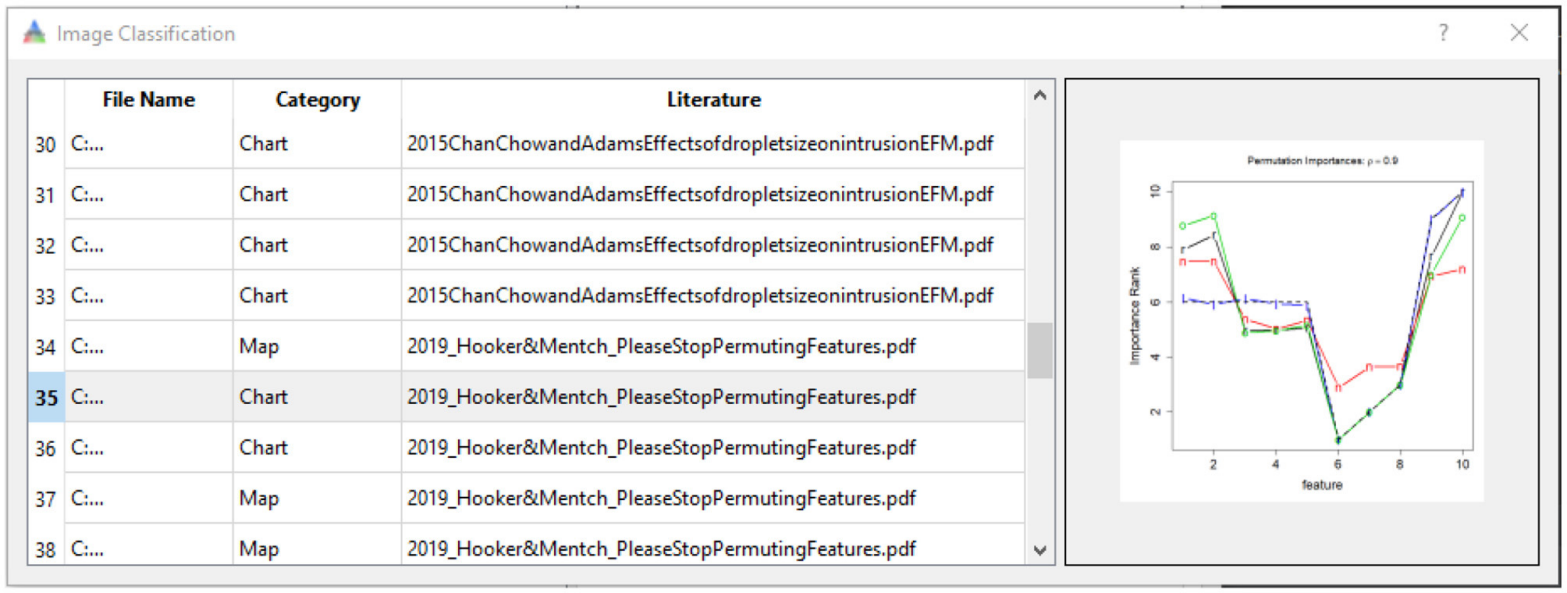
Data repository of labeled images created from unstructured knowledge products. The example shown is a simple binary classification with an accuracy of ~90%.

The model is available in the STA tool as part of the literature analysis workflow. The weights from the trained model are packaged with the tool and loaded on demand to classify extracted images. The STA user interface allows researchers to import (Figure 5) hundreds of unstructured knowledge products and rapidly receive a repository (Figure 6) of structured data to assist in geologic domain postulation.

## 3 Results

### 3.1 Binary classification

**Figure 7:**
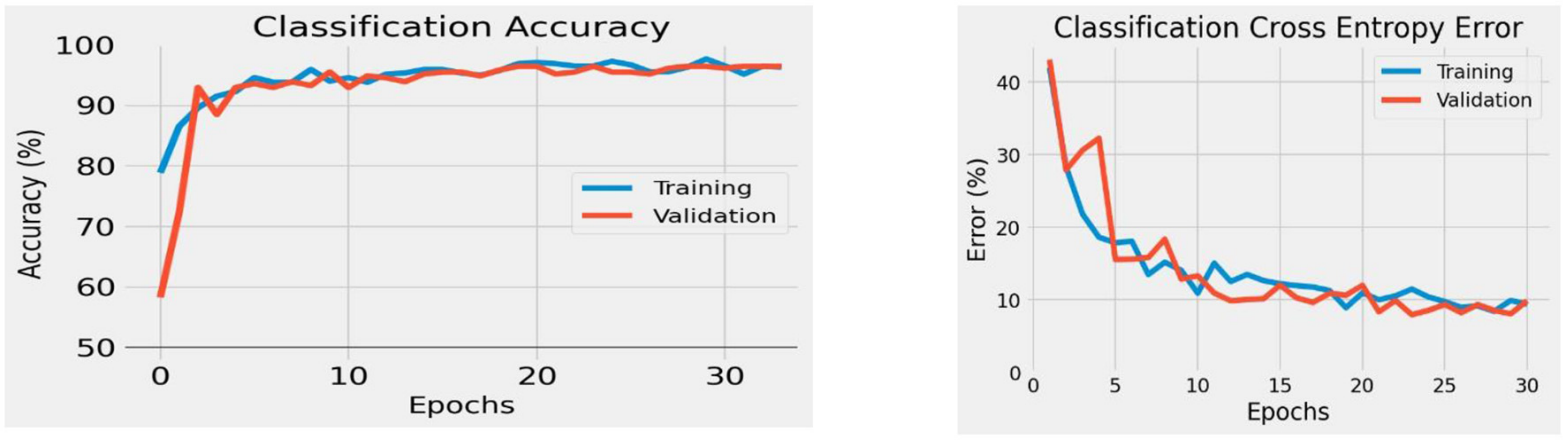
Classification accuracy **(left)** of the STA's image embedding tool during the training/testing (~30 epochs). Above 30 epochs overfitting occurred, which can be seen **(right)** with the convergence of cross entropy error.

**Figure 8:**
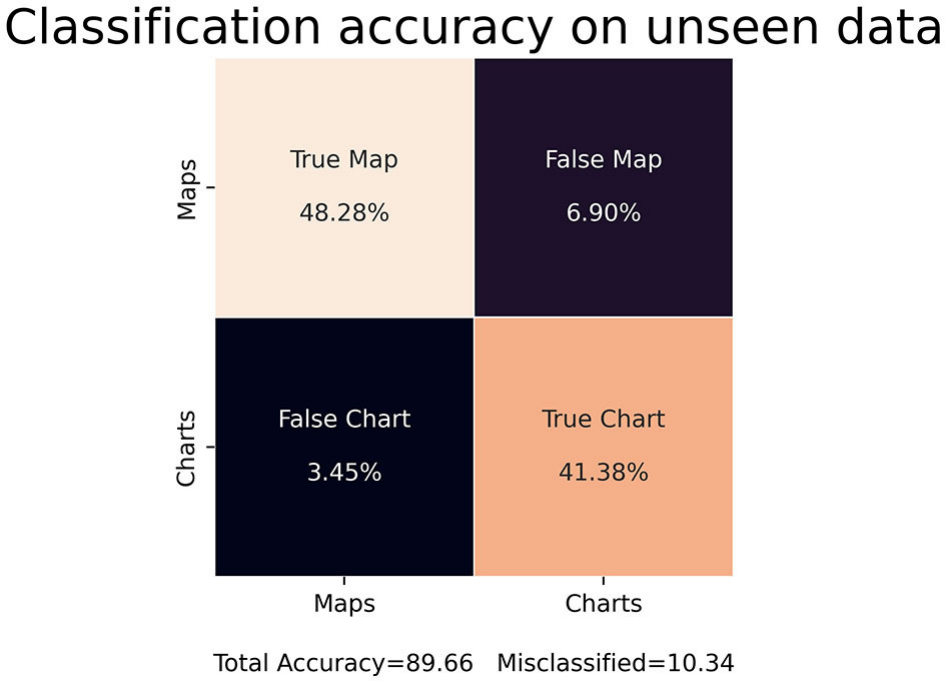
Classification accuracy on unseen data for the STA binary image embedding model. The model maintained an accuracy of roughly 90% on unseen data.

We applied the STA image embedding tool to geologic and geographic knowledge products about the northern Gulf of Mexico (GOM), which has been the use case for the entire development of the STA tool suite. Following binary training, the STA image embedding tool extracted images from the GOM and correctly labeled them with ~90 to ~95% accuracy for binary classification (Figure 7, left) with a loss entropy of <10% for ~30 to ~35 epochs (Figure 7, right). Beyond ~30 to ~35 epochs, the classification began to overfit and did not generalize to unseen datasets. For the binary classification, the image classification model maintained ~90% accuracy on unseen inputs, similar to the training/test split (Figure 8).

### 3.2 Multiclass classification

**Figure 9:**
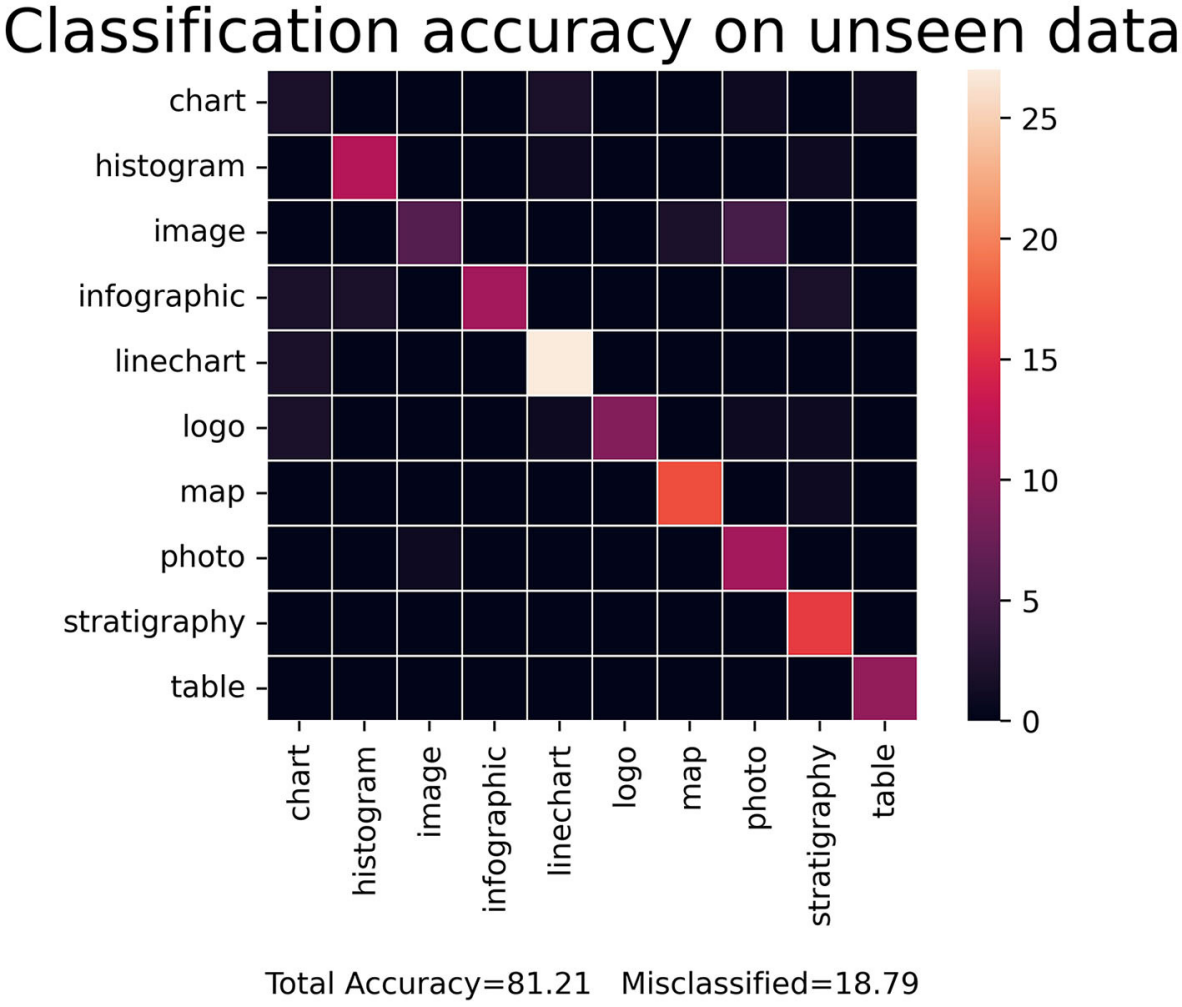
Accuracy matrix of the STA image labeling model expanded to 10 categories. While the accuracy has decreased, the model still accurately labels unseen data ~80% of the time.

When expanded beyond a binary classification, the STA image embedding tool still maintained a robust accuracy of ~86.67% on the validation dataset and 79.87% on the test dataset. The confusion matrix in Figure 9 shows the accuracy for each category in the multiclass model. The multiclass model begins to stagnate at 15 epochs and overfit after ~20 epochs. The metrics for 10 consolidated classes improved over the previous version with 14 classes, seeing accuracy in training change from 86.68 to 87.67% and accuracy in validation change from 84.25 to 86.67%. Additionally, it is worth noting that to achieve these results, the target size was increased to 256 from 64, as finer details for visually similar classes were causing incorrect predictions notable from the confusion matrix.

### 3.3 Classification speed

We tested the speed of the STA image embedding tool by importing a list of geological based knowledge products that were selected using NETL's Smart Search Tool (Rose et al., 2018). The tool was able to parse imagery from 1,000 documents and complete both the binary and multiclass models in <10 min.

## 4 Discussion

Image imbedding has been widely used in tasks, such as object proposal generation (Uijlings et al., 2013; Pont-Tuset et al., 2017; Zhang et al., 2017), tracking (Shrestha et al., 2020; Yang et al., 2021), object detection/recognition (Kohli et al., 2009; Juneja et al., 2013), and semantic segmentation (Noh et al., 2015; Wang et al., 2018). Despite such uses, rarely, if ever, has image imbedding been used to facilitate the literature review process.

The research presented in this paper envisions a space of AI and subject matter expert cooperation, where scientists utilize AI-informed models to assist in parsing literature and unstructured data like images, maps, cross sections, and other products to provide context to measured data (e.g., cores, well logs, seismic surveys). As part of the STA method, we presented a tool that allows researchers to more rapidly parse relevant geo-specific contextual knowledge to accelerate knowledge- and data-driven subsurface property analyses.

With a binary accuracy of ~90% and a multi-categorical accuracy of ~80% on unseen data sources, the STA image embedding tool can facilitate literature reviews, particularly in the geosciences, by parsing thousands of documents and images in a short time (<10 min).

From our personal experiences with the tool, we can attest to the image embedding tool's remarkable time-saving capabilities in the context of literature reviews. This categorization tool excels in swiftly parsing and labeling data, far outpacing human capabilities. As a result, we were able to efficiently review hundreds of images, bypassing the laborious process of sifting through thousands of pages of literature.

Nevertheless, while the STA Image Embedding tool holds promise, it is crucial to acknowledge its limitations. The tool is constrained by the categorization scope, confined to the categories explicitly discussed in the paper. Consequently, the STA Image Imbedding tool may struggle to accurately categorize images that diverge from these predefined categories (Wagner et al., 2022). Furthermore, the tool's accuracy is inextricably linked to the quality and availability of the training data it relies upon. In instances where training data is scarce or biased, the tool's capacity for accurate categorization may be compromised (Shorten and Khoshgoftaar, 2019). An additional limitation inherent, not just to this tool, but all image embedding, is its context-specific nature (Xu et al., 2019). This means that the tool's ability to categorize images accurately may be contextually bounded. For example, an image embedding tool trained on a dataset of medical images might not perform well when tasked with categorizing images of natural landscapes or other unrelated domains.

Overall, the results in this paper demonstrate the nascent potential for image embedding to help with literature reviews in a geospecific domain. Future research of the STA image embedding tool will include expanding the categorization and making it more specific to geological and geographic terms. In addition, current research of NETL's STA tool includes developing natural language processing (NLP) models to work in tandem with the image embedding to further facilitate knowledge discovery. We imagine that the NLP model will be able to improve image embedding accuracy by reading image figure labels and feeding that information to the CNN model. As well, we plan to receive user-specific feedback on the tool's capabilities and incorporate that feedback into future versions of the tool.

## 5 Conclusion

As AI continues to inform more high-stakes decisions (Bernabé-Moreno and Wildberger, 2019), a rich set of literature has been devoted to understanding how experts and AI can work together in various domains like education (Chen et al., 2020), health care (Han et al., 2020) supply chain economics (Toorajipour et al., 2022), human rights (Rodrigues, 2020), and software engineering (Hutchinson et al., 2021). As well, there has been substantial philosophical discussion regarding the “responsibility gap” associated with AI (i.e., determining culpability when AI systems fail) (Santoni de Sio and Mecacci, 2021).

At its core, this AI-expert-relationship delves into the nuanced dynamics of the AI-expert relationship, emphasizing the need for AI to augment decision-making without impeding or compromising the process (Bernabé-Moreno and Wildberger, 2019). With the research presented in this paper, we endorse the perspective that AI should inform decision-making, but not replace human expertise. Specifically, AI-enhanced tools, as exemplified in this paper, could enhance geo-specific research by parsing information about the heterogeneous physical environment. Researchers in the physical and earth sciences could be more easily informed on the essential attributes in an area of interest (AOI), allowing them greater insight during analysis. For instance, researchers investigating the Gulf of Mexico's natural-engineered energy system, including petroleum exploration, wave energy, and carbon storage, can gain valuable insights into the region's unique attributes. These insights include the Gulf of Mexico's susceptibility to hurricanes (Kossin et al., 2010), high sedimentation rates at the Mississippi River Delta Front (Chaytor et al., 2020), and distinctive geological features influenced by subsurface salt migration (Rowan et al., 2003). Understanding the Gulf of Mexico's unique climatic and geologic attributes equips researchers with the knowledge needed to make informed decisions regarding infrastructure in the region.

However, it is essential to recognize the limitations of AI. AI-informed insights are fundamentally reliant on the availability of data, which may not offer comprehensive coverage across entire subject areas, as the deepest understanding often resides with domain experts. Moreover, AI outputs are typically context-specific and my not be directly transferable to different conditions (Dunjko and Briegel, 2018). In contrast, human expertise is versatile and adaptable to various problems across different fields. Additionally, AI models often lack transparency and may be challenging for non-experts to understand (Yampolskiy, 2020). Consequently, subject matter experts are still essential for interpreting and validating AI-generated results.

In summary, while AI and ML are increasingly integral to high-stakes decision-making, they are most effective when they complement human expertise rather than replace it. The synergy between AI and expert knowledge allows for more informed and robust decision-making across a spectrum of domains, addressing both the potential and limitations of AI in guiding critical choices.

## Data availability statement

The original contributions presented in the study are included in the article/supplementary material, further inquiries can be directed to the corresponding author.

## Author contributions

BH: lead author and initial developer of methodology. DZ and MM-M: significant contributions to methodology and writing of all sections. PW: significant contributions to methodology and writing introductions and conclusions. AS: contributions to methodology development, review, and writing several sections. KR: significant contributions to method developments, writing, and mentorship. All authors contributed to the article and approved the submitted version.
